# Meta-inflammation in Hidradenitis suppurativa: from pathogenic evidence to therapeutic approaches

**DOI:** 10.3389/fimmu.2026.1827227

**Published:** 2026-05-25

**Authors:** Chiara Moltrasio, Abbas Khan, Namra Ahmad, Muhammad Adil Malik, Paola Maura Tricarico, Sergio Crovella, Abdelali Agouni, Angelo Valerio Marzano

**Affiliations:** 1 Dermatology Unit, Fondazione IRCCS Ca’ Granda Ospedale Maggiore Policlinico, Milan, Italy; 2 Department of Pharmaceutical Sciences, College of Pharmacy, QU Health, Qatar University, Doha, Qatar; 3 Department of Biomedical Sciences, Faculty of Medical and Life Sciences, Sunway University, Kuala Lumpur, Selangor, Malaysia; 4 School of Life Sciences, Central South University, Changsha, China; 5 The Third Xiangya Hospital, Central South University, Changsha, China; 6 Department of Pediatrics, Institute for Maternal and Child Health, IRCCS “Burlo Garofolo”, Trieste, Italy; 7 Department of Environmental and Prevention Science, University of Ferrara, Ferrara, Italy; 8 Department of Pathophysiology and Transplantation, Università degli Studi di Milano, Milan, Italy

**Keywords:** combination therapies, GLP-1 receptor agonist, Hidradenitis suppurativa, immunometabolism, metabolic syndrome, meta-inflammation

## Abstract

Hidradenitis suppurativa (HS) is a chronic, recurrent inflammatory skin disorder of the pilosebaceous unit, characterized by nodules, abscesses, and sinus tracts formation in apocrine gland-bearing skin. Increasing evidence suggests that HS is not solely a localized dermatological condition, but part of a broader systemic inflammatory state closely associated with metabolic dysfunction. Meta-inflammation, defined as a chronic, low-grade inflammatory response driven by metabolic imbalance, has emerged as a key mechanism linking obesity, insulin resistance, and immune dysregulation to the pathogenesis and progression of HS. It has indeed been demonstrated in numerous studies that HS and metabolic syndrome (MetS) share a core link through meta-inflammation, driven by common pro-inflammatory cytokines, such as tumor necrosis factor (TNF)-α, interleukin (IL)-1β and IL-6. These pathways, along with insulin resistance and obesity-related adipose tissue dysfunction, drive both skin disease severity and metabolic comorbidities often creating a self-perpetuating cycle of inflammation and tissue damage. Understanding the interplay between meta-inflammation and HS provides important insights into disease heterogeneity and highlights the need for a multidisciplinary therapeutic approach. Targeting meta-inflammation through lifestyle interventions, weight management, and immuno-metabolic therapies may complement established treatments and improve clinical outcomes. In this review, we explore the pathogenic link between meta-inflammation and HS and discuss the therapeutic targets arising from this intricate interplay.

## 1 Introduction

Hidradenitis suppurativa (HS) is a chronic relapsing autoinflammatory skin disorder with an average worldwide prevalence of 1%. It primarily affects follicular units within intertriginous areas, such as axillary, inguinal, and anogenital regions (1) and clinically manifesting with painful, deep-seated nodules and abscesses. In a certain proportion of patients, the disease course can lead to irreversible tissue destruction with multiple inflamed and pus-discharging interconnected tunnels and morbid scarring (1). This clinical manifestation accompanied by malodorous pus, pain and itch, significantly impacts the quality of life of patients, who often experience insomnia, anxiety, depression, and suicidal thoughts (2).

Despite its recent growing recognition, significant diagnostic delay persists in clinical practice, often preventing the timely intervention necessary to mitigate the disease’s debilitating sequelae (3). A state of dysregulation induces the disease itself through a wide array of immunological/inflammatory responses and mediators triggered by a strong genetic predisposition (4), environmental/epigenetic determinants, and hormonal/microbial factors (5).

Noteworthy, HS is increasingly regarded not merely as a dermatological entity but also as a systemic inflammatory condition (6), due to its significant association with a plethora of comorbid conditions, such as inflammatory bowel disease (IBD) (7), spondylarthritis (8), non-alcoholic fatty liver disease (NAFLD). -recently renamed metabolic dysfunction-associated steatotic liver disease (MASLD)- (9), metabolic syndrome (MetS) and related cardiovascular disease (10).

A significant association was observed between HS and MetS, which mainly encompasses central obesity, hypertension, dyslipidaemia and type 2 diabetes mellitus, metabolic phenotypes commonly found in HS patients (11). The patho-mechanisms linking these two conditions are mainly due to the role of adipose tissue that acts as an immunological organ, producing cytokines and mediators of systemic inflammation and metabolic control (12). The chronic low-grade inflammation triggered by metabolic perturbations is now referred to as “meta-inflammation” (13–16) and it is increasingly recognized as a key HS amplifier (17).

This scenario is giving rise to novel promising immunometabolic strategies to be included in the therapeutic arsenal for HS.

This review directs attention toward the relevance of meta-inflammation in HS and describes the potential of immunometabolic therapies that support holistic management strategies addressing both cutaneous inflammation and underlying metabolic alterations.

### 1.1 Dissecting the pathogenesis: from follicular occlusion to systemic inflammation

The pathogenesis of HS implies a complex process that extends beyond a simple mechanical dysfunction of the hair follicle and concludes in a profound systemic inflammatory state (5).

In the early stages of lesion development, occlusion and cystic expansion of the hair follicle promote the release – from damaged cells - of keratin debris, damage-associated molecular patterns (DAMPs) and trapped bacteria (pathogen-associated molecular patterns, PAMPs) that act as a potent trigger for immune cell infiltration (18). This damage in the expanded hair follicle provokes a robust inflammatory context in which innate immune cells (neutrophils, macrophages, dendritic cells, and complement), adaptive T-cellular (T helper -Th-1, Th17) and B-cellular immune reactions finally leads to pus formation, tissue destruction and epidermal thickening, in which a critical role is also played by IL-36 (5, 19). This cytokine milieu is further amplified by the surrounding hypertrophic adipose tissue, which itself functions as an active endocrine organ, secreting additional inflammatory mediators and dysregulated adipokines (20).

Critically, the disease process is not limited to the skin. The persistent inflammatory state is maintained by systemic factors, including alterations in both cutaneous and gut microbiome, which in turn are implicated in priming the innate immune system, although their precise role is still being defined (21). The progression from an occluded follicle to widespread inflammation, abscess formation, and scarring is thus a cascade in which a local insult ignites a pre-existing systemic inflammatory predisposition.

This comprehensive understanding of HS as a systemic immune-mediated inflammatory disorder provides the foundation for elucidating its extensive range of comorbid conditions.

## 2 The systemic burden and comorbidities of HS

### 2.1 HS phenotypes and associated comorbid conditions

HS is increasingly recognized as a systemic inflammatory disease, as underscored by its strong association with comorbidities that significantly impact patient health and mortality (22).

Over the past few decades, several attempts have been made to classify the various HS subtypes (23–28). Each of the proposed phenotypes is based on clinical cutaneous characteristics, including the anatomical distribution and qualitative differences of skin lesions. However, there is still no consensus on the optimal categorization and there is a lack of validation studies based on inter-rater reliability (IRR), especially in individual patient assessments (29). Interestingly, Jørgensen et al. (30) reported six HS subtypes taking into account comorbidity patterns and inflammatory/metabolic blood signatures: i) female, ii) familial, iii) diabetes, iv) psychiatric disease, v) Crohn’s disease, and vi) asthma. In this study, inflammatory arthritis and hypertension were more related to familial subtype. The diabetes subtype showed a tendency toward comorbid diabetes and hypertension, as well as chronic obstructive pulmonary disease (COPD) and osteoarthritis. The highest levels of systemic inflammation were observed in the Crohn’s disease and diabetes subtypes, while the female subtype exhibited the lowest levels of inflammation. Additionally, the diabetes subtype had the highest blood glucose and triglyceride levels, whereas the lowest levels were found in the female subtype. Furthermore, both a high body mass index (BMI) and smoking may influence disease severity and phenotype. Obesity has also been more frequently associated with the frictional furuncle and regular HS phenotypes.

### 2.2 The intertwined relationship between HS and metabolic syndrome

Among the most significant associations with HS is that with MetS (31), defined as a constellation of abnormalities, including hypertension, dyslipidemia, insulin resistance, and central obesity, that collectively increase the risk of serious health issues like cardiovascular disease and type 2 diabetes mellitus (32).

Multiple studies confirmed that individuals with HS are significantly more likely to develop MetS compared to the general population (even after adjusting for confounding factors such as age and gender) (33–35), with odds ratios (ORs) ranging from approximately 1.82 to 2.12 (26). Of note, hospital-based studies (often representing more severe cases) reported even higher odds of MetS (up to 3.89-fold) (36).

HS is specifically linked to higher rates of MetS components, including obesity, diabetes, hypertension and dyslipidaemia, as demonstrated by a recent study in which the authors found that HS patients had nearly three times the odds of developing obesity and hypertension (17). A broader meta-analysis in 2024 reported HS patients have significantly higher odds of type 2 diabetes mellitus (OR ≈ 2.78), obesity (OR ≈ 2.48), and smoking (OR≈ 3.10), reinforcing the clustering of HS with metabolic risk factors (37).

The link between HS and MetS components is not coincidental but is driven by shared underlying complex and multifactorial pathophysiological mechanisms (38, 39). The relationship appears to be a vicious cycle, in which the chronic inflammation from HS may increase the risk for the individual components of MetS, and conversely, the inflammation associated with MetS exacerbates the cutaneous manifestations and HS chronicity, a central mechanism called meta-inflammation (31). The strong clinical association between metabolic dysfunction, obesity, and HS severity is also well-documented. Obesity is identified as a key predisposing factor, and various studies have shown that a higher body mass index (BMI) is a risk factor for developing HS and is associated with a more severe disease presentation (40). Recently, Mintoff et al. (41) emphasized that lifestyle and genetic factors, combined with obesity-driven meta-inflammation, exacerbate HS severity, underlying the need to address obesity and associated metabolic abnormalities as part of comprehensive HS management.

### 2.3 The adipose tissue as an endocrine and immunological organ and metabolic consequences

The immunological mechanisms linking obesity and HS are mainly guided by adipose tissue (42), now recognized as an active endocrine organ that produces and secretes a plethora of pro-inflammatory cytokines and adipokines such as leptin, resistin, and visfatin that contribute to local and systemic inflammation (43).

Adipose tissue inflammation is triggered and sustained by dysfunctional adipocytes, that secrete inflammatory adipokines, and by infiltration of bone marrow-derived immune cells, particularly M1 macrophages and T-lymphocytes, that send signals through the production of cytokines and chemoattractant mediators (42). More in detail, under metabolic stress conditions, such as obesity, hypertrophic adipocytes and tissue resident immune cells undergo phenotypic changes, initiating the secretion of inflammatory adipokines and cytokines that act both locally and systemically to induce peripheral insulin resistance. The inflammatory reaction is sustained by C-C chemokine receptor type 2 (CCR2), monocyte chemoattractant protein (MCP), and semaphorin 3A (SEMA3A). Additionally, pro-inflammatory cytokines such as tumor necrosis factor (TNF)-α, IL-1 β and IL-6, promote energy expenditure, - defined as the total amount of energy an individual uses to maintain essential body functions-, and mediate, together with interferon (IFN)-y, the effects of adipose tissue inflammation on distant organs (44). Generally, postprandial macrophage-derived IL-1β promotes adipose tissue remodeling by stimulating adipogenesis and enhancing insulin secretion, thereby channeling glucose toward macrophages (45, 46). However, persistently elevated IL-1β levels in obesity contribute to adipose tissue inflammation (47) and favor myeloid lineage skewing, ultimately sustaining systemic inflammation (48).

Regarding IL-6, it is interesting to note that the cell source of this interleukin in the adipose tissue determines its effects: while adipocyte-derived IL-6 promotes adipose tissue macrophage recruitment, myeloid cell-derived IL-6 inhibits this process (49). These opposite actions involve a switch of IL-6 signaling from a canonical mode to a noncanonical *trans*-signaling mode, thus demonstrating that the source of IL-6 production plays a major role in the physiological regulation of metabolism (49). Additionally, IL-6 plays an important role in regulating metabolism also upon exercise: during exercise, circulating IL-6 increases insulin secretion and delays gastric emptying, thereby reducing postprandial blood glucose levels (50, 51). Moreover, IL-6 inhibits fatty acid esterification in adipose tissue, thereby counteracting obesity (52, 53). Therefore, it is important to mention that, although IL-6 upregulation is a component of obesity-related systemic inflammation, exercise induced-IL-6 confers a metabolism regulation with a wide array of protective functions.

The chronic inflammatory state, as also seen in HS, involves immune cells and dysfunctional adipose tissue, thus creating a pathogenic cross-activation loop, where macrophages and other immune cells within hypertrophic fat deposits secrete substantial amounts of pro-inflammatory cytokines, dominantly IFN-y and TNF-α. The latter directly interferes with insulin receptor signaling pathways, promoting insulin resistance and establishing a direct biological link to the development of MetS (54). Mechanistically, chronic inflammation and insulin resistance reciprocally reinforce one another; indeed, TNF-α can impair insulin signaling, while metabolic dysfunction primes immune cells for pro-inflammatory phenotypes, with enhanced glycolysis and reduced regulatory controls (55). Furthermore, insuline resistance is promoted by mechanistic Target of Rapamycin (mTOR), a central metabolic regulator that also enhances inflammatory cytokines production (56).

IFN-y also acts as a master regulator of the adipose inflammatory cascade, stimulating macrophages and T cells to adopt a pro-inflammatory (M1) phenotype and its deficiency has been shown to reduce inflammatory gene expression in white adipose tissue and improve insulin sensitivity in obese mouse models (57, 58).

Alongside this, decreased levels of anti-inflammatory adiponectin have been detected in HS patients, suggesting that unbalanced adipokine levels may directly contribute to HS onset and severity (59).

The leading role of adipose tissue, acting as a key driver of systemic inflammation, elevates the management of metabolic health from a supportive measure to a foundation of effective HS therapy. The cumulative inflammatory burden from this interplay also significantly increases the risk of major adverse cardiovascular events (MACE), including myocardial infarction, stroke, and cardiovascular-related mortality (60). In this regard, a large population-based cohort study reported that the incidence rate of MACE was significantly elevated in HS patients when compared to healthy controls and individuals diagnosed with psoriasis. This elevated risk is likely caused by persistent systemic inflammation with elevated TNF-α and IL-6 circulating levels, promoting atherogenesis, thereby increasing the likelihood of acute cardiovascular events. Furthermore, the cytokine landscape typical of HS contributes to endothelial dysfunction, oxidative stress, and vascular remodeling, key processes involved in cardiovascular alterations (61).

Consequently, a comprehensive multidisciplinary treatment strategy needs to be essentially integrated, incorporating aggressive inflammatory control with proactive management of weight, metabolic parameters, and routine cardiovascular risk assessment to mitigate both cutaneous and systemic disease manifestations.

## 3 Main therapeutic strategies targeting meta-inflammation beyond dietary restriction

To address the metabolic drivers of meta-inflammation, physicians and researchers are paying increasing attention to drugs traditionally reserved for diabetes and obesity. At the same time, it remains of central importance for HS management smoking cessation and dietary restriction/modulation, as substantial weight loss can trigger disease remission by reducing mechanical friction and adipose-derived pro-inflammatory cytokines. Indeed, weight-loss therapies, particularly the mediterranean diet, the ketogenic and the carbohydrate-reduced diets, have shown promising results in reducing disease flare-ups (62).

### 3.1 Insulin sensitization

Metformin, the first-line drug for type 2 diabetes mellitus, is one of the most studied immunometabolic agents in HS, proving effective in a considerable number of cases, especially in metabolically dysregulated HS phenotypes (63, 64). While its mechanism of action in HS still needs to be fully clarified, it has been demonstrated that it exerts an anti-inflammatory and anti-metabolic effect through the following main mechanisms of action: i) improvement of insulin sensitivity; ii) activation of 5’ adenosine monophosphate-activated protein kinase (AMPK), an enzyme that activates glucose and fatty acid uptake and oxidation when cellular energy is low; iii) inhibition of mTOR signaling, that play a key role in cell growth and metabolism with environmental inputs including nutrients and growth factors; iv) reduction of IL-6, TNF-α and IL-1β circulating levels as well as Th17 polarization; the latter through inhibition of STAT3 via mammalian target of rapamycin complex 1 (mTORC1) (65, 66).

Overall, it has been proved that metformin can mediate its anti-inflammatory and anti-metabolic effects in HS by influencing the cell energy metabolism, thus providing a scientific rationale for targeting cellular metabolism in HS to counteract inflammation (67).

Also from a clinical perspective, different studies showed the successful use of metformin in HS patients. As an example, a retrospective study found metformin to be effective and well tolerated in all HS patients treated (63), while a prospective study reported both clinical and quality of life improvement in > 30% of patients with HS taking metformin (64), thus suggesting the valid addition of metformin in the therapeutic armamentarium of HS (68).

Noteworthy, a recent multicenter, randomized, double-blind, placebo-controlled study (69) demonstrated that the clinical efficacy of metformin in conjunction with doxycycline is no greater than that of doxycycline monotherapy. However, the improvements observed in metabolic parameters may provide a rationale for prescribing metformin in combination with other anti-inflammatory therapies for HS, especially in patients with known metabolic risk factors.

### 3.2 Glucagon-like peptide-1 receptor agonists

Glucagon-like peptide-1 (GLP-1) receptor agonists (GLP-1-RAs), also known as GLP-1 analogs, originally developed for the management of type 2 diabetes, are gaining prominence in managing obesity and metabolic-related conditions in HS (70–76). GLP-1 is an incretin hormone produced and secreted by intestinal enteroendocrine L-cells and certain neurons within the nucleus of the solitary tract in the brainstem upon food consumption (77). Endogenous GLP-1 is rapidly degraded by dipeptidyl peptidase-4 (DPP-4) and renal clearance, resulting in a half-life of approximately 2 minutes. GLP-1-RAs are GLP-1 analogs with a slower elimination kinetics compared to native GLP-1 and resistant to DPP-4 degradation (78). GLP-1-RAs primarily increase insulin synthesis in response to hyperglycaemia (78) and contribute to weight loss by regulating energy balance in the human hypothalamus (79), along with cardioprotective and vasodilatory effects (80).

Currently, liraglutide, semaglutide, and tirzepatide are food and drug administration (FDA)-approved for obesity treatments and are also emerging as promising therapeutic strategies for inflammatory skin disorders such as HS (81).

Case reports of HS (82, 83) and a prospective proof-of-concept study (84) demonstrated clinical benefits of liraglutide, in terms of reduction in mean Hurley stage, dermatology life quality index (DLQI) and flares. Experimental studies, conducted on human primary chondrocytes (85) and mouse obese diabetic mice with psoriasiform skin (86), demonstrated that, beyond weight reduction, liraglutide seems to play an important role in improving inflammation through the inhibition of TNF-α and other pivotal pro-inflammatory cytokines such as IL-17 and IL-23 as well as the suppression of nuclear factor κB (NF-κB) activity (85–87). In diabetic patients with atherosclerosis, liraglutide also negatively impacts monocyte chemotactic protein 1 (MCP-1) and L-selectin secretion, thus blocking the directional migration and activations of monocytes and macrophages (88).

Good clinical outcomes have also been reached with semaglutide. Lyons and colleagues (89) performed a retrospective study on the use of semaglutide in 30 HS patients, in which a significant reduction in mean DLQI, patient-reported flares and metabolic markers was reported. Although the authors underlined its usefulness as adjunctive treatment of HS, further randomized clinical trials are needed to assess its efficacy and safety.

Regarding tirzepatide, only a case report has been published so far, showing a marked reduction in abscesses and inflammatory nodules count, alongside a decrease in DLQI score (90).

While liraglutide, semaglutide and tirzepatide are the only GLP-1 analogs currently used in HS management, other GLP-1-RAs such as dulaglutide, exenatide and albiglutide may also represent promising future adjuvant therapies for HS (75).

In conclusion, although data are still scarce, GLP-1-RAs seem to represent a promising therapeutic intersection between metabolic (weight loss, improvement of insulin resistance and modulation of inflammatory phenotype of macrophages) and inflammatory control (reduction of pro-inflammatory cytokines systemic levels and potential effects on the gut microbiome).

### 3.3 Cytokine blockage with metabolic implications

#### 3.3.1 Tumor necrosis factor

TNF-α is a key pro-inflammatory cytokine that play an important role in adipose tissue functions, especially in obesity and metabolic disease. It is well-know that it is involved in inflammation acting as a crucial player, in immune regulation, in cell survival and apoptosis and metabolic signaling. Adalimumab, in particular, is the first FDA-and European Medicine Agency (EMA)- approved biologic for HS (91) and considered among the first-line options in the medical management of moderate-to-severe disease (92, 93). Besides being crucial mediator of inflammation, TNF also exerts significant metabolic control. It interferes with insulin signaling by i) increasing serine phosphorylation of insulin receptor substrate (IRS), ii) decreasing glucose transporter type 4 (GLUT4) expression and iii) inhibiting insulin receptor (94). TNF also promotes lipolysis, - by increasing the release of free fatty acids (FFA) from the adipose tissue to the liver, which further exacerbate insulin resistance and promote steatohepatitis (95–97) – and suppresses adipogenesis by inhibiting the differentiation of preadipocytes into mature adipocytes and the expression of peroxisome proliferator-activated receptor gamma (PPAR-γ), thus promoting a dysfunctional fat tissue remodeling (98).

Overall, TNF promotes the metainflammation through the maintenance of inflammatory loop in adipose tissue and its blockage may ameliorate both cutaneous and systemic manifestations of HS.

#### 3.3.2 IL-1β and NLRP3 inflammasome

IL-1β is a central upstream cytokine linking metabolic dysfunction to chronic cutaneous inflammation in HS. In metabolically driven diseases such as obesity and insulin resistance, IL-1β acts as a key bridge between innate immune activation and Th17/IL-17 amplification. It is also considered as the core meta-inflammatory amplifier because it increases C reactive protein (CRP) through IL-6 induction, promotes insulin secretion and alters adipokines balance, thus creating a self-perpetuating metabolic-immune loop (44). It is well known that the secreted form of IL-1 β is produced from its inactive form through NLR family pyrin domain containing 3 (NLRP3) inflammasome-mediated caspase cleavage (99–101). NLRP3 inhibitors such as MCC950 analogs have been shown in ex vivo models to reduce the release of IL-1β, TNF-α, IL-17A and other pro-inflammatory mediators, highlighting the interplay between metabolic signals and immune activation (102). From a therapeutic point of view, MCC950 is the best-studied NLRP3 inhibitor; it is highly specific and doesn’t inhibit other inflammasomes or related-pathways (103). Of note, Petrasca et al. (67) through an inflammasome assay, demonstrated that metformin inhibits IL-1β to a similar extent as MCC950, thus confirming that metformin also blocks inflammation by interfering with NLRP3 inflammasome activity, as previously demonstrated by Tsuji et al. (104). While MCC950 is in the pre-clinical stage for HS, other NLRP3 inhibitors such as OLT1177 (dapansutrile) (105), tranilast (106) and β-hydroxybutyrate (107) are showing promising results for other indications and may have therapeutic benefit in HS, modulating metabolic inflammation.

## 4 Emerging immunometabolic therapies: future perspectives

### 4.1 SGLT2 inhibitors

While biologic therapies effectively target downstream immune-inflammatory pathways, durable disease modification may require upstream metabolic intervention. Sodium–glucose cotransporter-2 (SGLT2) inhibitors, commonly used for type 2 diabetes and cardiorenal protection, may represent a promising, albeit underexplored, therapeutic avenue within the immunometabolic framework of HS.

Beyond glycemic control, SGLT2 inhibitors reduce hyperinsulinemia, visceral adiposity, oxidative stress and systemic levels of inflammatory mediators (108). Experimental and clinical data in cardiometabolic populations demonstrated attenuation of TNF-α, IL-6, and inflammasome activation (109) —pathways also implicated in HS pathogenesis. By lowering circulating insulin levels, SGLT2 inhibition may indirectly reduce mTOR signaling pathway (110), a proposed contributor factor to follicular hyperkeratosis and immune amplification in HS. This upstream modulation distinguishes SGLT2 inhibitors from cytokine-directed biologics and aligns with emerging models of dual-axis therapy targeting both metabolic drivers and inflammatory mediators.

In this evolving landscape, the term “next-generation SGLT2 inhibitors” is emerging to describe contemporary SGLT2 therapies that go beyond glucose reduction (108). However, clinical evidence in HS remains absent. Prospective observational studies and randomized controlled trials comparing SGLT2 inhibitors with established metabolic therapies (e.g., metformin or GLP-1-RAs) are needed.

Notably, safety considerations warrant careful study, particularly in HS patients with perineal involvement. Indeed, Fournier’s gangrene, vulvovaginal candidiasis, balanitis and genital fungal infections may occur after SGLT2 inhibitors administration (111, 112), thus underscoring the importance of vigilant patient selection and monitoring.

With the field evolving toward integrated immunometabolic management, SGLT2 inhibitors may offer a biologically plausible strategy to reduce systemic inflammatory state while improving cardiometabolic risk. Future research should determine whether metabolic pathway modulation can translate into sustained dermatologic remission and improved long-term outcomes in this complex disease, taking into account the risks listed above.

### 4.2 Microbiome-targeted therapies

HS is increasingly understood as a microbiome–immune–metabolic disorder, where both skin and gut dysbiosis contribute to chronic inflammation, sinus tract formation, and systemic comorbidities (113). Although the exact role of dysbiosis in HS pathogenesis remains to be clarified, different studies have demonstrated a reduction in cutaneous microbial diversity in HS patients, characterized by an increase in the abundance of anaerobic and opportunistic bacteria and a reduction in commensal species (114, 115). The gut microbiome has not yet been thoroughly studied, but recent evidence reported a reduced intestinal microbiota diversity and a shift toward a pro-inflammatory state, characterized by an increase in bacteria such as *Bilophila wadsworthia* and *Ruminococcus gnavus* and *callidus*, and a decrease in anti-inflammatory, short-chain fatty acid (SCFA)-producing bacteria like *Faecalibacterium prausnitzii*, *unclassified Clostridiales, unclassified Firmicutes, and Fusicatenibacter* (116). SCFA-producing bacteria are crucial for maintaining gut homeostasis and immune regulation and their reduction leads to a decrease in anti-inflammatory metabolites, thus promoting a pro-inflammatory environment. Additionally, the gut microbiome in HS patients shows an overrepresentation of specific metabolic pathways, such as those involved in D-glucarate and D-galactarate degradation, which are associated with exacerbated systemic inflammation (116–119). This gut dysbiosis increases gut permeability, triggering robust systemic inflammation with an increased levels of pro-inflammatory cytokines such as TNF-α and IL-17; the adipose tissue is strongly linked to this axis, being the major source of pro-inflammatory mediators, which interact with the gut-derived inflammation (120).

In this evolving context, microbiome-targeted therapies may be an emerging strategy to address meta-inflammation in HS affecting the gut–skin–adipose inflammatory axis.

First of all, it has been discussed the potential utility of probiotics, whose mechanism of actions mainly involve: i) augmentation of SCFA production, particularly, butyrate; ii) strengthening of the intestinal barrier and iii) reduction of endotoxemia (121, 122). A promising strategy seems to involve the topical application of probiotics containing commensal species such as *Corynebacterium* spp., *Staphylococcus epidermidis*, and *Cutibacterium acnes* to protect against local cutaneous inflammatory events (122). However, probiotic application could be preceded by an antibiotic regimen to eradicate Gram-negative anaerobic bacteria, such as *Prevotella* and *Porphyromonas*, which dominate the HS microbiome (121).

Also, prebiotics aim to nourish beneficial gut and skin bacteria, potentially reducing the inflammation and dysbiosis linked to the disease. While direct research on prebiotics for HS is limited, it has been reported that these agents increase butyrate production, improve insulin sensitivity and reduce systemic levels of TNF-α, potentially improving HS severity via metabolic modulation (114).

Another promising experimental approach lies on fecal microbiota transplantation (FMT). While specifically limited data exists on its direct efficacy for HS, two clinical trials are currently investigating FMT and capsule-based transplantation to reduce inflammatory responses in HS patients. A phase II trial (NCT04924270) (123) is currently testing weekly FMT for 4 weeks in patients with chronic inflammatory diseases, including 20 with HS, while a pilot study (NCT06058520) (124) is investigating capsule-based microbiome transplantation for HS, to demonstrate the central hypothesis that oral microbiota transplant therapy (MTT) alters the gut microbiome, thus influencing cutaneous microbiota via systemically absorbed gut-derived metabolites.

All these novel potential strategies, together with other emerging approaches such as topical anti-biofilm agents, laser-assisted biofilm disruption and surgical excision of biofilm-rich tracts deserve future attention for their inclusion as combination therapies alongside biologics/small molecules and/or other conventional therapies.

## 5 Conclusions

HS is increasingly conceptualized within a meta-inflammatory disease framework, where metabolic dysfunction (obesity, insulin resistance, dysregulated adipokines) provides a chronic pro-inflammatory milieu and innate/adaptive immune dysregulation drives persistent cutaneous inflammation, reinforcing each other. The vicious cycle between systemic metabolic stress and local inflammatory mechanisms perpetuates disease activity and progression, thus supporting holistic management strategies that address both cutaneous inflammation and underlying metabolic factors.

As thoroughly discussed, there is a robust association between HS and metabolic dysfunction, mediated by shared inflammatory pathways and dysregulated immunometabolism.

From a clinical point of view, initial (and periodic) screening is therefore recommended for all patients with HS (even in young patients) to identify any metabolic dysfunction, primarily by assessing BMI, waist circumference, blood pressure, lipid profile, glycated hemoglobin, fasting plasma glucose, liver enzymes and inflammatory markers such as C-reactive protein (CRP) and fasting insulin. Consequently, the stratification of the phenotype is equally important, as follows: i) predominantly metabolic HS (obesity and/or insulin resistance; mild to moderate inflammatory burden); ii) mixed inflammatory-metabolic HS (moderate to severe disease; obesity or diabetes) and iii) predominantly inflammatory HS (severe disease without obvious metabolic abnormalities).

From a therapeutic perspective, different promising approaches such as GLP-1-RAs are emerging.

However, randomized trials specifically targeting meta-inflammation in HS are still limited and further research is needed to explore the efficacy and safety of emerging immunometabolic therapies. In addition, better biomarkers are needed to stratify HS patients based on metabolic phenotype and predict response to immunometabolic therapies. In this regard, the metabolic consequences of chronic inflammation are reflected in metabolomics and lipidomics. Preliminary evidence demonstrated that plasma metabolomic profiling of HS patients showed a transition in the non-enzymatic antioxidant defense that is centered on cysteine metabolism, including alterations in cysteine-related metabolic pathways, depletion of specific oxylipins, and reduction of PPAR signaling. These findings imply a complex interorgan metabolic interaction, in which energy metabolism and inflammation are closely interconnected, thus providing a scientific rationale for future investigations into metabolic targets for HS management (125).

Similarly, plasma lipidomic analysis indicated an alteration in circulating oxylipins related to HS activity and severity, including hydroxyeicosatetraenoic (HETE) and dihydroxyeicosatrienoic acid (DHET) (126). The understanding of systemic metabolic profile is therefore essential to understanding the strict interplay between immune-mediated inflammation and metabolic alteration underlying HS. Moreover, integrating microbiomics into an overall -omic framework will offer a comprehensive view of how microbial metabolite production (e.g. SCFAs) and dysbiosis affect, respectively, the host’s systemic immunity and ability to metabolize drugs.

Overall, targeting meta-inflammation represents a compelling mechanistic and therapeutic paradigm in HS, warranting further clinical investigation. Notably, future paradigms may also combine biologic cytokine blockade, GLP-1-RAsand insulin sensitizers. This dual-axis targeting immune and metabolic pathways may provide superior disease control.
